# Implementing 360-Degree Simulation Training During Psychiatry Placement Inductions: A Mixed Methods Training Evaluation

**DOI:** 10.1192/bjo.2022.86

**Published:** 2022-06-20

**Authors:** Seri Abraham, Thomas Hewson, Emily Kaye, Niksa Soltani, Gareth Preston, Ankur Khanna, Sara Higgins, Roshelle Ramkisson

**Affiliations:** 1 Pennine Care NHS Foundation Trust, Greater Manchester, United Kingdom; 2 Manchester Metropolitan University, Manchester, United Kingdom; 3 University of Bolton, Bolton, United Kingdom; 4 University of Manchester, Manchester, United Kingdom; 5 Lancashire and South Cumbria NHS Foundation Trust, Preston, United Kingdom

## Abstract

**Aims:**

The authors designed a simulation training programme for foundation doctors beginning psychiatry placements across a large mental health trust. The simulation training aimed to improve the confidence, competence, and well-being of foundation doctors through exposing them to realistic psychiatry scenarios and teaching clinical skills in a safe environment.

**Methods:**

Four clinical scenarios were filmed with a 360-degree camera, professional actress, and doctors working in psychiatry. The scenarios depicted the journey of a patient being admitted onto a psychiatry ward from the community. Various clinical skills were embedded into the videos including psychiatric history taking, risk assessment, managing acute distress, managing comorbid physical and mental health problems, using the Mental Health Act, and teamwork with colleagues. All videos were delivered to learners using simulation with head-mounted-displays (HMDs). Each video lasted 6–8 minutes and was accompanied by pre-briefing and de-briefing with experienced psychiatrists for a further 15–20 minutes. Participants rated their confidence regarding several skills in psychiatry on Likert scales from 1 to 5 immediately before and after the session. Wilcoxon signed rank tests were conducted to detect statistically significant differences in learner's median confidence ratings before and after the training. Free-text questions explored trainee's most and least favourite aspects of the simulation. A survey also was distributed to learners 2-months after the training to assess how it had influenced their clinical practice.

**Results:**

20 foundation doctors completed the training and provided feedback. Following the simulation training, there were statistically significant improvements in foundation doctor's confidence in: completing psychiatric assessments (p < 0.01), managing physical health problems in psychiatry (p < 0.05), managing acute distress (p < 0.01), reporting information to senior colleagues (p < 0.05), and containing anxiety when communicating with patients (p < 0.05). Trainees highlighted the debriefing, group discussions, and “interactive” simulation videos as the most useful aspects of the training. Some trainees enjoyed viewing the 360-degree videos, whilst others found the HMDs difficult to use. Of the 8 trainees who completed feedback 2 months after the training, 7 (87.5%) felt that it had helped them in their current roles. All trainees agreed (37.5%) or strongly agreed (62.5%) that the simulation scenarios were closely aligned to real-life clinical encounters.

**Conclusion:**

Simulation training in psychiatry using 360-degree videos and HMDs is generally well-received amongst foundation doctors. Embedding simulation training into placement induction can improve the confidence and skills of junior doctors starting psychiatry placements.

